# Successful Termination in a Case of Normal Pregnancy

**Published:** 1856-10

**Authors:** Edwin R. Maxson

**Affiliations:** Geneva, N. Y.


### SUCCESSFUL TERMINATION IN A CASE OF NORMAL PREGNANCY, COMPLICATED WITH EXTRA-UTERINE.

BY EDWIN R. MAXSON, M. D., OF GENEVA, N. Y.

I was called, September 8th, 1856, to attend Mrs. ——, aged about thirty-five, in her third confinement.

On inquiry, she stated to me that her health had been good; and, as her pains were regular, she was delivered, in a few hours of a fine female child, which weighed eight pounds.

I observed nothing unusual in her condition or symptoms at the time, except that the placenta adhered slightly, at or near the left fallopian tube, or its orifice.

This adhesion, however, was slight, as it was detached by a gentle twisting motion given to that portion of the placenta which presented, thus rendering it unnecessary to introduce the hand into the womb for its detachment.

On examination of the placenta to see if any portion had been left, I discovered, at the part which I supposed adhered, an appearance which indicated that it had been attached at that point in a manner different from the ordinary attachment, the part appearing as if it had been torn from something to which it had adhered very tenaciously.

But, as no portion of it appeared to have been left, I passed the matter by, without any further concern at the time; and, as she appeared quite well, I left, and saw no more of her till the following day, September 9th, at which time, all her symptoms being good, I discharged her, not intending to visit her again.

On the 11th of September, three days after her confinement, the husband consulted me in relation to a tumor, which he said had given her some inconvenience, and more or less pain, during the six or eight preceding months.

He represented the tumor as being the size of a hen's egg, and occupying the left iliac region; and that it was then giving her some uneasiness. He assured me that she had never suffered from hernia, and promised to notify me if it gave her any serious inconvenience.

I was called, September 12th, four days after her confinement, in great haste, and found that she had been suffering the most excruciating pain, in the left iliac region, and as she thought, in the tumor itself. She described the pain as being the most severe she had ever suffered, and lasting for twenty or thirty minutes, and then suddenly subsiding, leaving only a slight uneasiness in the part.

The tumor, too, had disappeared suddenly, with the pain; and as she had become quite easy, I had a flannel wet in warm camphorated spirits applied; and as she had no chill or fever, I left, without any further prescription, with directions to notify me, should the tumor again make its appearance, or the pain return.

On the following day, September 13th, a slight contraction of the womb (or what she called a severe after-pain) brought away a perfectly formed fœtus, as described to me by the mother of the lady, about two and a half inches in length, and about the size of the thumb. She examined it with a neighboring lady, with great care, and assured me that it was perfectly formed, but that it felt very hard.

During the same pain, there was also expelled what was probably the placenta, which she described as a circular piece of flesh, about the size of a dollar, though somewhat thicker. This they also examined carefully, and washed it in water, in order to be sure that it was not coagulated blood, and were thus sure that it was flesh, as they called it.

I need hardly say, that the character of the ladies in question is such, that their statement in this matter is entirely reliable.

Now, here was a case in which a normal pregnancy was carried out, with a fallopian pregnancy occurring at the same, or some previous time; the fallopian fœtus, with its placenta, being expelled from the fallopian tube, into the womb, doubtless, during the severe pain which occurred the fourth day after her confinement, and expelled from the womb the fifth day, as above described.

I do not know whether a case similar to this has ever occurred or been noticed before.

The fallopian fœtus, in this case, with its placenta, must have occupied that portion of the tube nearest the womb; and I suspect the two placentas adhered to each other, at the mouth of the tube; and that the slight twist which I gave the uterine placenta, to detach it, detached or loosened the fallopian placenta and fœtus, and thus probably favored their expulsion into the womb, as it occurred the fourth day after, instead of the tubes bursting and their being expelled into the peritoneal sac, as is usual in such cases.

She does not remember to have noticed the tumor previous to her last pregnancy; but thinks it had been visible for the last six or eight months before her confinement; thus rendering it probable that the uterine and fallopian conceptions, occurred at, or nearly at the same time.

The left fallopian tube, which evidently contained the fœtus in this case, must have contracted very soon after its expulsion of the fœtus, to nearly its natural size; as there has, since the severe paroxysm of pain alluded to, been no appearance of a tumor in the left iliac region; and the lady is now, September 25th, quite well.—*Western Lancet*

